# Evodiamine Induces Transient Receptor Potential Vanilloid-1-Mediated Protective Autophagy in U87-MG Astrocytes

**DOI:** 10.1155/2013/354840

**Published:** 2013-12-24

**Authors:** Ann-Jeng Liu, Sheng-Hao Wang, Sz-Ying Hou, Chien-Ju Lin, Wen-Ta Chiu, Sheng-Huang Hsiao, Thay-Hsiung Chen, Chwen-Ming Shih

**Affiliations:** ^1^Graduate Institute of Clinical Medicine, College of Medicine, Taipei Medical University, Taipei, Taiwan; ^2^Department of Neurosurgery, Taipei City Hospital Ren-Ai Branch, Taipei, Taiwan; ^3^Department of Biochemistry, School of Medicine, Taipei Medical University, 250 Wu-Hsing Street, Taipei 110, Taiwan; ^4^Graduate Institute of Medical Sciences, College of Medicine, Taipei Medical University, Taipei, Taiwan; ^5^Department of Neurosurgery, Taipei Municipal Wan-Fang Hospital, Taipei, Taiwan; ^6^Department of Surgery, College of Medicine, Taipei Medical University, Taiwan; ^7^Division of Cardiac Surgery, Cathy General Hospital, Taipei, Taiwan; ^8^Traditional Herbal Medicine Research Center, Taipei Medical University Hospital, Taipei, Taiwan

## Abstract

Cerebral ischemia is a leading cause of mortality and morbidity worldwide, which results in cognitive and motor dysfunction, neurodegenerative diseases, and death. Evodiamine (Evo) is extracted from *Evodia rutaecarpa* Bentham, a plant widely used in Chinese herbal medicine, which possesses variable biological abilities, such as anticancer, anti-inflammation, antiobesity, anti-Alzheimer's disease, antimetastatic, antianoxic, and antinociceptive functions. But the effect of Evo on ischemic stroke is unclear. Increasing data suggest that activation of autophagy, an adaptive response to environmental stresses, could protect neurons from ischemia-induced cell death. In this study, we found that Evo induced autophagy in U87-MG astrocytes. A scavenger of extracellular calcium and an antagonist of transient receptor potential vanilloid-1 (TRPV-1) decreased the percentage of autophagy accompanied by an increase in apoptosis, suggesting that Evo may induce calcium-mediated protective autophagy resulting from an influx of extracellular calcium. The same phenomena were also confirmed by a small interfering RNA technique to knock down the expression of TRPV1. Finally, Evo-induced c-Jun N-terminal kinases (JNK) activation was reduced by a TRPV1 antagonist, indicating that Evo-induced autophagy may occur through a calcium/c-Jun N-terminal kinase (JNK) pathway. Collectively, Evo induced an influx of extracellular calcium, which led to JNK-mediated protective autophagy, and this provides a new option for ischemic stroke treatment.

## 1. Introduction

Brain ischemia, a restriction of the blood supply to tissues, causing a shortage of oxygen and glucose needed for cellular metabolism, is the leading cause of death and disability worldwide. Tissue plasminogen activator (tPA) therapy is the major treatment for ischemic stroke. However, the window for tPA treatment is within 0~3 h after onset of a stroke, which limits its clinical use. Accumulating evidence demonstrating that protective autophagy is induced in a stroke mode since blockade of autophagy results in an increase in cell death [1] suggests an agent possessing autophagy-inducing ability which may provide a new option for ischemic stroke treatment.

Evodiamine (Evo) is a quinozole alkaloid isolated from *Evodia rutaecarpa* Bentham which is widely used in Chinese herbal medicine with variable effects, such as anticancer, anti-inflammation, anti-obesity, anti-Alzheimer's disease, antimetastatic, antianoxic, and anti-nociceptive functions [2]. An *in vitro* study showed that Evo has an endothelium-dependent vasodilatory effect on isolated rat mesenteric arteries [3]. Furthermore, Evo may serve as an antiatherosclerosis agent since it can inhibit oxidative stress-induced production of chemokine receptor (CCR)-1, CCR2, and intracellular adhesion molecule (ICAM)-1 [4, 5], suggesting that Evo may possess anticardiovascular disease activity. It was reported that Evo protected rats from myocardial ischemia-reperfusion injury [6]. However, the effect of Evo on ischemic stroke in the brain is not well understood.

Evo is considered a transient receptor potential vanilloid-1 (TRPV1) agonist [7]. TRPV1, a ligand-gated calcium ion channel activated by vanilloids, protons, and various environmental stresses, was implicated as a pain-sensing transducer and plays a key role in regulating cell death. Capsaicin, a well-known TRPV1 agonist, extracted from hot chili peppers, was shown to lead to human nasopharyngeal carcinoma cell death through mitochondrial and endoplasmic reticular (ER) stress [8]. Furthermore, capsaicin induced apoptosis in glioma cells through a p38-mediated signal pathway [9]. However, capsazepine (CPZ), a TRPV1 antagonist potentiated the anticancer effect of tumor necrosis factor-related apoptosis-inducing ligand (TRAIL) through upregulation of death receptors [10]. Therefore, the role of TRPV1 in regulating apoptosis is controversial, and the effect of TRPV1 on autophagy needs to be investigated.

Autophagy, an evolutionarily conserved mechanism regulating the turnover of long-lived proteins and damaged organelles, is also considered type II programmed cell death. Autophagy is characterized by the formation of double-membrane vesicles (autophagosomes) and the processing of microtubule-associated protein 1 light chain 3 (LC3). Dysfunction of autophagy may lead to cancer development, bacterial and viral infections, neurodegenerative disorders, and cardiovascular diseases [11, 12]. The role of autophagy in ischemia has been widely investigated. Rapamycin, an autophagy inducer, protected against hypoxia-induced brain injury through induction of autophagy [13]. Superoxide dismutase (SOD)-2 knockdown led to ischemic brain damage under a hyperglycemic condition through induction of oxidative stress, which was associated with a reduction in autophagy [14]. Furthermore, melatonin-induced neuronal protection against cell death resulting from glucose-oxygen deprivation was abolished by the autophagy inhibitor, 3-methyladenine (3-MA) [15]. All of the above suggest that autophagy may play a protective role in ischemia-induced neuronal damage.

Human U87-MG astrocytes (obtained from a Caucasian strain) classified as grade IV glioblastoma were employed as a cell model in this study. We found that Evo induced protective autophagy of astrocytes. Treatment with a TRPV1 antagonist was able to increase intracellular calcium level and block Evo-induced c-Jun N-terminal kinase (JNK) activation which results in suppression of autophagy. Combined with our previous report [16], we demonstrated that Evo induced an increase in intracellular calcium resulting from an influx of extracellular calcium through the TRPV1 channel, which led to JNK-mediated protective autophagy.

## 2. Materials and Methods

### 2.1. Cell Culture, Treatment, and Chemicals

Human U87-MG astrocytes (obtained from a Caucasian strain) classified as grade IV glioblastoma were purchased from the American Type Culture Collection (Manassas, VA) and grown at 37°C in culture medium consisting of Dulbecco's modified Eagle's medium (DMEM) supplemented with 10% heat-inactivated fetal bovine serum (FBS), 200 mM L-glutamine, 100 U/mL penicillin, 100 *μ*g/mL streptomycin, 100 mM sodium pyruvate, and 1% nonessential amino acids. The mixture was kept in a humidified atmosphere containing 5% CO_2_. For the drug-response experiments, cells were either pretreated (treatment group) or not (control group) with the indicated inhibitor for 1 h and then incubated with 6 *μ*M Evo for the rest of the experimental period [16]. DMEM, FBS, and nonessential amino acids were purchased from Hyclone (Logan, UT), and phenol red-free RPMI, L-glutamine, penicillin-streptomycin, and sodium pyruvate were obtained from Gibco (Grand Island, NY). Evo, bovine serum albumin (BSA), acridine orange (AO), Fluo-3 AM, 5,5,6,6,-tetrachloro-1,1,3,3,-tetraethylbenzimidazolylcarbocyanine iodide (JC-1), ethylene glycol tetra-acetic acid (EGTA), CPZ, and 3-(4,5-dimethylthiazol-2-yl)-2,5-diphenyltetrazolium bromide (MTT) were purchased from Sigma (St. Louis, MO). Propidium iodide (PI) was purchased from Calbiochem (San Diego, CA). The Annexin-V-FITC reagent was supplied by Biovision (Mountain View, CA). Rabbit polyclonal anti-LC3 was obtained from MBL International (Nagoya, Japan). Rabbit polyclonal anti-GAPDH and anti-TRPV1 were obtained from Cell Signaling (Beverly, MA). Secondary anti-bodies, including horseradish peroxidase- (HRP-) conjugated goat anti-rabbit immunoglobulin G (IgG), were purchased from Pierce (Rockford, IL). Polyvinylidene difluoride (PVDF) membranes were supplied by Millipore (Bedford, MA), and the Protein Assay Dye Reagent was from Bio-Rad (Hercules, CA).

### 2.2. Measurement of Acidic Vesicular Organelles (AVOs)

Autophagy was analyzed by flow cytometry with AO dye according to published procedures [17]. After the indicated treatment, cells were stained with AO (1 *μ*g/mL) for a period of 20 min. Trypsinized adherent cells and cells suspended in the medium were collected in phenol red-free RPMI medium. Green (510~530 nm) and red (650 nm) fluorescence emissions from 10^4^ cells illuminated with blue (488 nm) excitation light were measured on a flow cytometer using CellQuest software (Becton Dickinson, San Jose, CA). The percentage of autophagy was summed across the upper-left and upper-right quadrants.

### 2.3. Measurement of Apoptosis

Apoptosis was evaluated by flow cytometry using two-color analysis of FITC-labeled annexin V/PI doublestaining, as described in our previous publication [18]. Trypsinized adherent cells and suspended cells in the medium were collected in HEPES buffer containing 10 mM HEPES (pH 7.4), 140 mM NaCl, and 2.5 mM CaCl_2_. Subsequently, cells were stained with annexin V (1 *μ*g/mL) and PI (0.2 ng/mL) for 15 min and then analyzed on a flow cytometer using CellQuest software (Becton Dickinson). The percentage of total apoptosis was the sum of primary apoptosis (annexin V+/PI−) and late apoptosis (annexin V+/PI+).

### 2.4. Measurement of Intracellular Calcium

After treatment, astrocytes were harvested and incubated with 500 nM Fluo-3 AM dye for 30 min at 37°C and then were immediately analyzed on a flow cytometer (530 nm) using FL-1 as a detector. Relative intracellular calcium concentrations were calculated from the ratio of the geometric mean values of the FL-1 peak generated from Evo-treated cells against each respective control, as indicated in the figure legends.

### 2.5. Western Blot Analysis

Adherent cells and suspended cells were collected and lysed with 50 *μ*L of lysis buffer containing 25 mM HEPES, 1.5% Triton X-100, 0.1% sodium dodecyl sulfate (SDS), 0.5 M NaCl, 5 mM EDTA, 0.1 mM sodium deoxycholate, and a protease inhibitor cocktail (Roche, Boehringer Mannheim, Germany) [19]. We then added sampling buffer (60 mM Tris-HCl at pH 6.8, 2% SDS, 10% glycerol, and 140 mM *β*-mercaptoethanol) to each lysate, and the mixture was sonicated using Microson Ultrasonic Cell Disruptor (Misonix Inc, Farmingdale, NY), subsequently boiled for 7 min, and centrifuged at 15,000 g for 5 min. The amount of protein was determined using the Bio-Rad Protein Assay Dye Reagent. Proteins electrotransferred onto PVDF membranes were immunoblotted with anti-LC3, anti-GAPDH, and anti-TRPV1 anti-bodies. Detection was performed with appropriate HRP-conjugated secondary anti-bodies and enhanced chemiluminescence reagent (Pierce). The band density was quantified by Gel-Pro Analyzer densitometry software (Media Cybernetics, Silver Spring, MD).

### 2.6. Knockdown of TRPV1

Astrocytes were transfected with 50 nM of small interfering (si)RNA using the Lipofectamine RNAiMAX reagent (Invitrogen, San Diego, CA) according to the manufacturer's instructions. In brief, cells were incubated with lipofectamine RNAimax reagent and 50 nM TRPV1 siRNA (Santa Cruz Biotechnology, Santa Cruz, CA) for 6 h, and then the medium was refreshed, followed by incubation for a further 42 h. Transfected cells were treated with Evo for the indicated time and analyzed by flow cytometry with AO staining or annexin V/PI double-staining.

### 2.7. Statistical Analysis

Values are expressed as the mean ± standard deviation (SD). Statistical analysis was performed using Student's *t*-test (for two groups) or a one-way analysis of variance (ANOVA) followed by Duncan's multiple-range test (for three or more groups). *P* values of <0.05 were considered statistically significant.

## 3. Results

### 3.1. Evo Triggers Calcium-Mediated Protective Autophagy in Astrocytes

**Figure 1:**
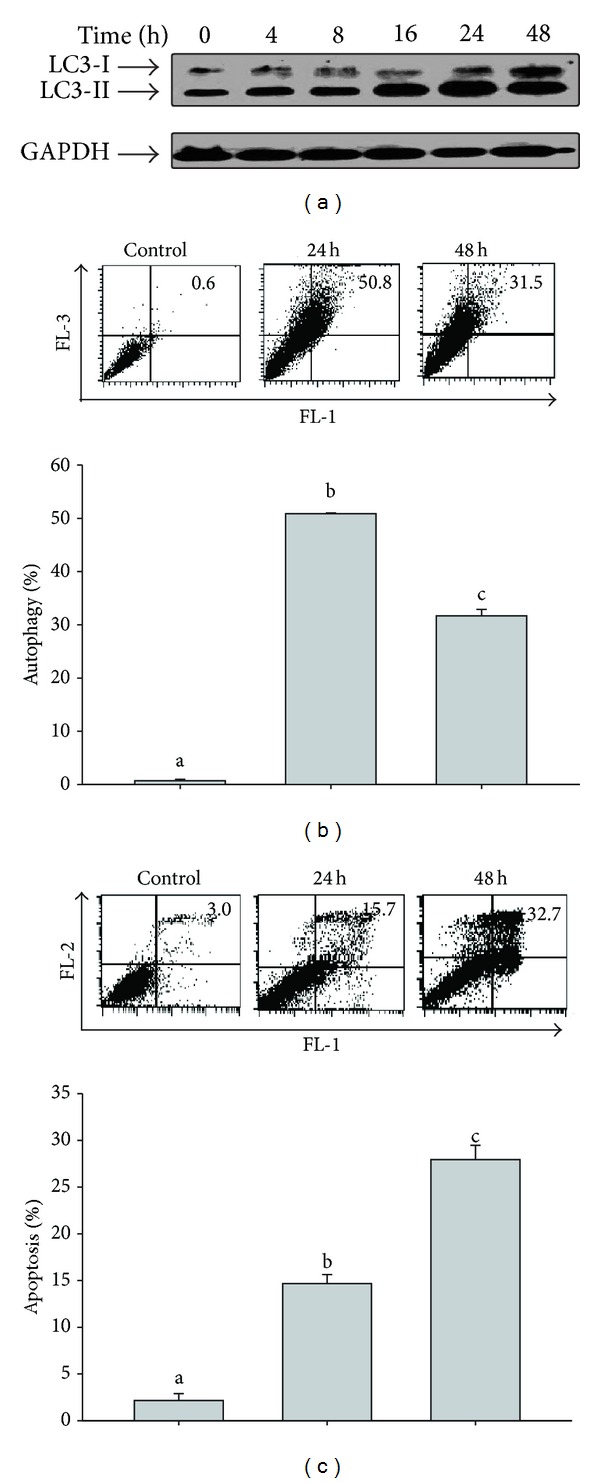
Evodiamine (Evo) induces autophagy and apoptosis in astrocytes. Cells were treated with 6 *μ*M of Evo for the indicated time and then analyzed by Western blotting with anti-LC3 and anti-GAPDH anti-bodies. GAPDH was used as an internal control to normalize the amount of proteins applied in each lane (a). Cells were treated with 6 *μ*M of Evo for 24 or 48 h, using flow cytometry with acridine orange staining for autophagy (b). Data presented in the upper panel represent the results of three independent experiments, and the respective statistical results are presented in lower panels of (b). (c) Cells were treated with 6 *μ*M Evo for the indicated time periods and then analyzed by flow cytometry with annexin-V/PI double-staining for apoptosis. Data presented in the upper panel represent the results of three independent experiments, and the respective statistical results are presented in lower panels of B. One-way ANOVA followed by Duncan's multiple-range test was used to determine whether the results for the experimental groups significantly differed from those of the respective controls. Columns not sharing the same superscript significantly differ (*P* < 0.05).

To investigate the effect of Evo in astrocytes, immunoblotting was applied to detect variations in LC3, a hallmark of autophagy. As demonstrated in Figure 1(a), the protein level of LC3-II increased after treatment with Evo for 16 h. Furthermore, flow cytometry with AO dye and annexin V/PI double dye was, respectively, applied to determine levels of autophagy and apoptosis. As shown in Figure 1(b), the percentage of cells containing autophagosomes increased and reached a maximum of 52% at 24 h. But the percentage of cells undergoing apoptosis (indicated in the upper-right quadrant) increased in a dose-dependent manner and reached 27% after 48 h of treatment with Evo (Figure 1(c)).

**Figure 2:**
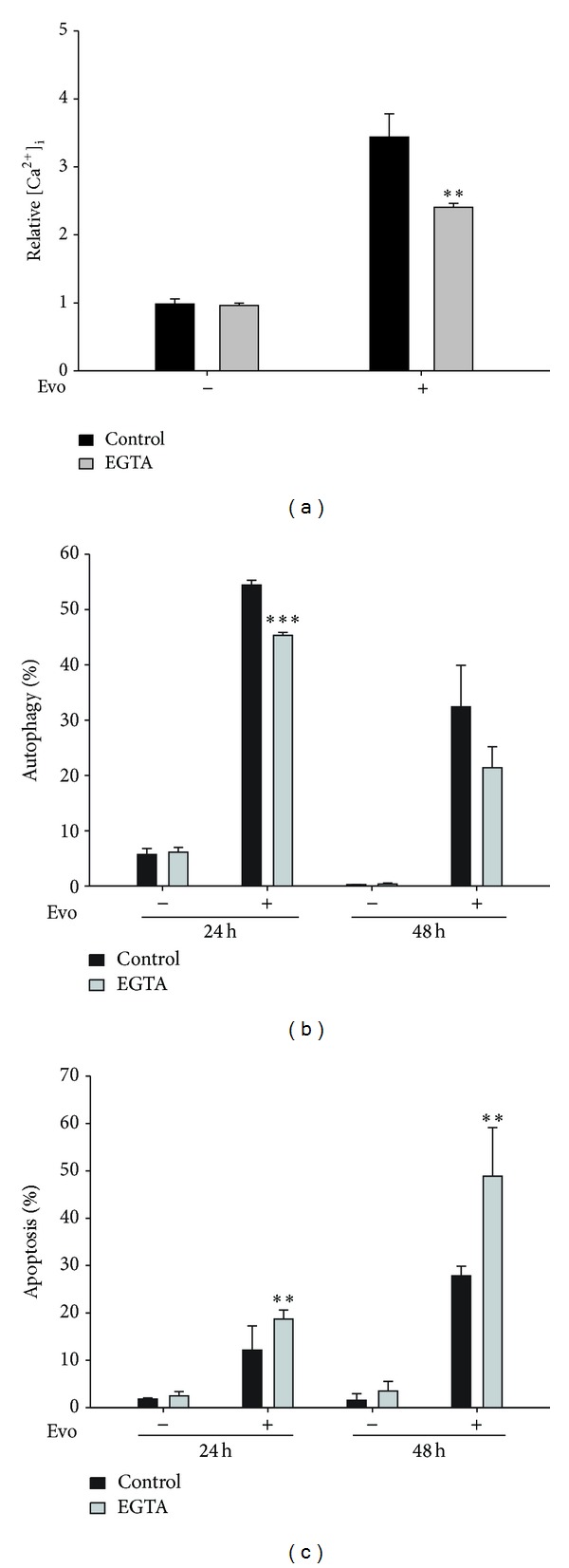
Evodiamine (Evo) induces calcium-mediated protective autophagy in astrocytes. (a) Cells were pretreated with EGTA, a scavenger of extracellular calcium, for 1 h, and then incubated with Evo for a further 8 h. Levels of intracellular calcium were measured by flow cytometry with Fluo-3 AM dye. The effects of EGTA on autophagy (b) and apoptosis (c) were evaluated by flow cytometry using acridine orange staining and annexin V/PI dye, respectively. **P < 0.01, ***P < 0.001 compared to the respective control, by Student's *t*-test.

Our previous report demonstrated that Evo induced release of calcium from the ER, which led to induction of autophagy and apoptosis. However, the role of extracellular calcium in Evo-induced autophagy is unclear. In this study, a scavenger of extracellular calcium (EGTA) was used to detect the involvement of extracellular calcium. Using flow cytometry with Fluo-3 AM dye, we investigated the variation in intracellular calcium. As shown in Figure 2(a), Evo induced an elevation in intracellular calcium at 8 h, which increased 3.1-fold compared to the control. Cells pretreated with EGTA were resistant to Evo, suggesting that an influx of extracellular calcium was involved in the Evo-induced increase in intracellular calcium. Furthermore, EGTA also reduced Evo-induced autophagy accompanied by an increase in apoptosis (Figures 2(b) and 2(c)). These results indicated that Evo induced elevation of intracellular calcium resulting from an influx of extracellular calcium, which led to the formation of protective autophagy against apoptosis.

### 3.2. Evo Induces TRPV1-Mediated Autophagy

**Figure 3:**
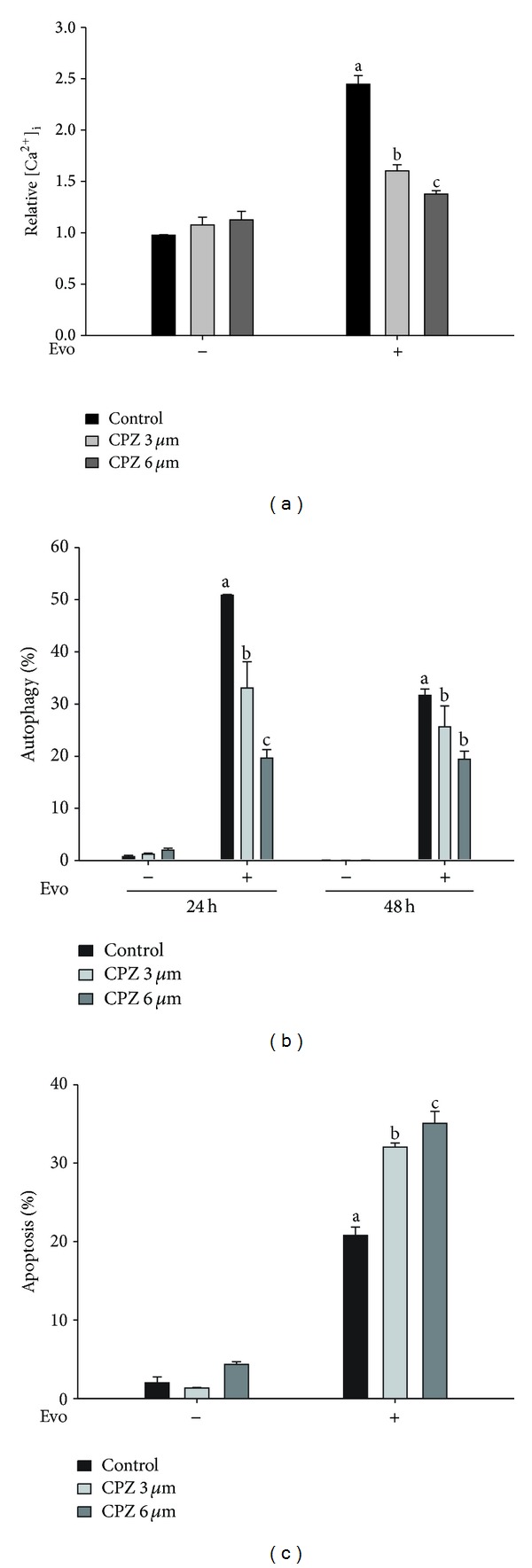
Evodiamine (Evo) induces TRPV1-dependent protective autophagy in astrocytes. (a) Cells were pretreated with capsazepine (CPZ), a TRPV1 antagonist, for 1 h, and then incubated with Evo for a further 8 h. Levels of intracellular calcium were measured by flow cytometry with Fluo-3 AM dye. (b) The effects of CPZ on autophagy were evaluated by flow cytometry using acridine orange staining. (c) Cells pretreated with CPZ were incubated with Evo for 48 h. The percentage of apoptosis was determined using annexin V/PI double staining on a flow cytometer. One-way ANOVA followed by Duncan's multiple-range test was used to determine whether the results for the experimental groups significantly differed from those of the respective controls. Columns not sharing the same superscript significantly differ (*P* < 0.05).

TRPV1 is a ligand-gated calcium ion channel, which plays a key role in regulating cell death. It was reported that Evo can activate the TRPV1 channel, but the effect of TRPV1 on autophagy is unclear. In this study, we investigated the role of TRPV1 in Evo-induced autophagy. As indicated in Figure 3(a), the Evo-induced increase in intracellular calcium was reduced by treatment with a TRPV1 antagonist (CPZ) in a dose-dependent manner. Furthermore, the percentage of cells that underwent autophagy was reduced in CPZ-treated astrocytes (Figure 3(b)). In contrast, Evo-induced apoptosis was further enhanced by treatment with CPZ at 48 h (Figure 3(c)), suggesting that Evo-induced calcium-dependent protective autophagy may have been due to activation of the TRPV1 channel.

**Figure 4:**
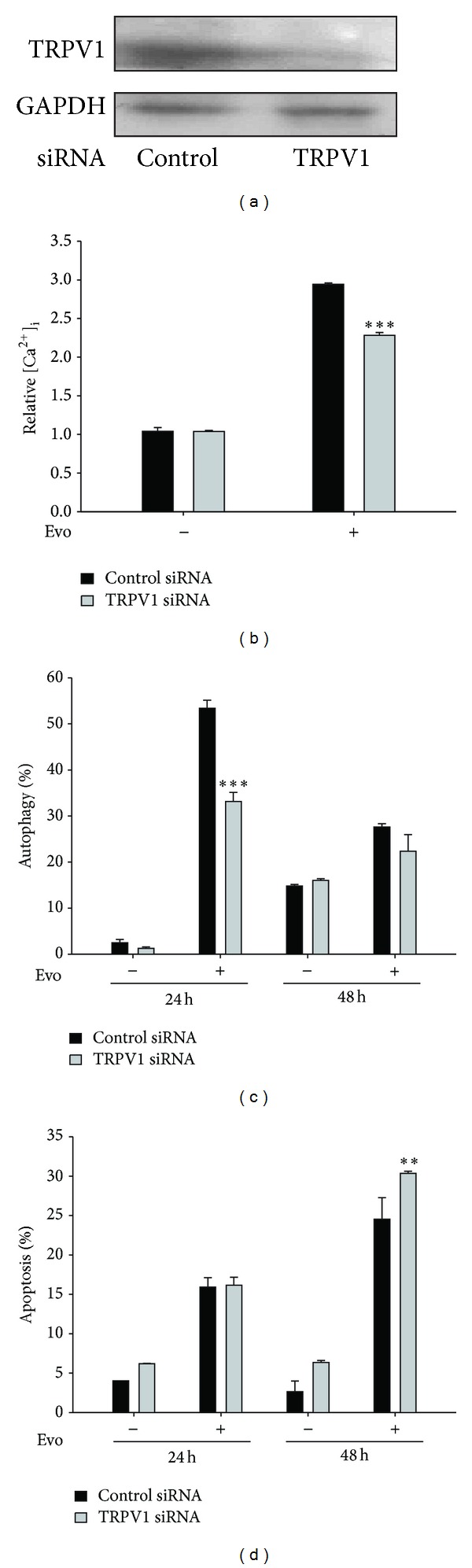
Reduction of evodiamine-(Evo)-induced protective autophagy by silencing the TRPV1 gene. (a) Cells were transfected with control or TRPV1 siRNA using the lipofectamine RNAimax reagent for 48 h, and the level of TRPV1 protein was measured by an immunoblot assay using anti-TRPV1 and anti-GAPDH anti-bodies to monitor the efficiency of the siRNA. GAPDH was used as an internal control to normalize the amount of proteins applied in each lane. (b) The elevation of cytosolic calcium induced by Evo was reduced by knockdown of TRPV1. Cells with or without knockdown of TRPV1 were treated with Evo for 8 h and then analyzed by Fluo-3 AM staining using flow cytometry. After transfection, cells were treated with Evo for another 24 or 48 h, trypsinized, and collected to determine percentages of autophagy (c) and apoptosis (d), respectively, using acridine orange staining and annexin V/PI staining. **P < 0.01, ***P < 0.001 compared to the respective control, by Student's *t*-test.

To further confirm the role of TRPV1 in Evo-induced autophagy, cells were treated with siRNA against the TRPV1 coding sequence. Using immunoblotting, we observed that levels of the TRVP1 protein were significantly reduced after being transfected with TRPV1 siRNA (Figure 4(a)). TRPV1 knockdown resulted in reductions in levels of intracellular calcium and autophagy after treatment with Evo (Figures 4(b) and 4(c)). However, the percentage of apoptosis further increased in cells with TRPV1 knockdown after treatment with Evo (Figure 4(d)). Collectively, these findings suggest that Evo-induced protective autophagy in astrocytes is mediated by the TRPV1 signaling pathway.

### 3.3. Evo Induces TRPV1-Mediated JNK Activation

**Figure 5:**
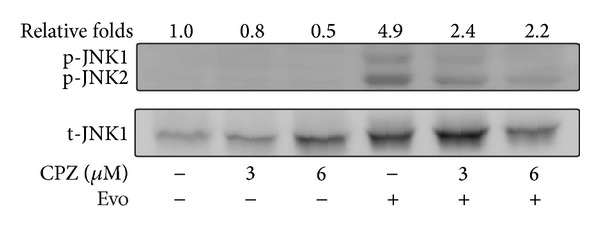
Evodiamine (Evo) induces TRPV1-mediated JNK activation. Cells treated with or without capsazepine (CPZ) were incubated with Evo for a further 12 h. Cell lysates were analyzed using Western blotting with anti-p-JNK and anti-t-JNK1 anti-bodies. t-JNK1 was used as an internal control to normalize the amount of proteins applied in each lane. The phosphorylated JNKs were quantified using Gel-Pro Analyzer densitometry software.

In our previous report, we found that Evo could induce JNK-mediated protective autophagy since blockade of JNK resulted in an increase in apoptosis [16]. Furthermore, Evo-induced JNK activation resulted from an increase in intracellular calcium released from the ER. However, the role of extracellular calcium was not mentioned. In this study, using a TRPV1 antagonist, we examined the role of TRPV1 in Evo-induced JNK activation. As demonstrated in Figure 5, Evo-induced JNK activation was reduced after treatment with CPZ, indicating that Evo-induced JNK activation was affected by both calcium released from the ER and an influx of extracellular calcium.

## 4. Discussion

Ischemic stroke, a leading cause of mortality and morbidity worldwide, leads to severe cognitive and motor dysfunction, neurodegenerative diseases, and death [20]. The only therapy for acute cerebral ischemia is tPA treatment within a 1~3 h time window after onset of a stroke. Therefore, identifying novel therapeutic targets is a challenge in this field. In this study, we found that Evo induced protective autophagy in astrocytes. Furthermore, a scavenger of extracellular calcium resulted in a reduction in autophagy and an increase in apoptosis. An inhibitor of TRPV1 also suppressed Evo-induced autophagy and intracellular calcium elevation accompanied by an increase in apoptosis, suggesting that Evo-induced protective autophagy may have resulted from extracellular calcium influx through the TRPV1 channel. The same phenomena were also confirmed using an siRNA technique against TRPV1. Finally, inhibition of TRPV1 by treatment with CPZ decreased Evo-induced JNK activation. Collectively, Evo induced an influx of extracellular calcium through the TRPV1 channel, which subsequently led to JNK activation and induction of protective autophagy.

TRPV1 is expressed by astrocytes, and Evo induced a TRPV1-dependent increase in intracellular calcium, which was responsible for JNK-mediated protective autophagy. Consistent with our results, capsaicin induced reactive oxygen species (ROS)/AMPK-mediated protective autophagy in thymocytes [21]. Furthermore, capsaicin also enhanced hepatic PPAR*δ* and autophagy-related proteins to reduce hepatic inflammatory factor in wild-type but not TRPV1−/− mice [22], suggesting that TRPV1 activation may induce survival autophagy. However, it was reported that capsaicin induced cell cycle arrest and apoptosis in urothelial bladder cancer cells through TRPV1 activation [23]. In this report, we observed that Evo induced apoptosis in a time-dependent manner, which was not reduced by treatment with CPZ. Moreover, blockade of calcium release from the ER by treatment with an IP_3_R channel inhibitor reduced Evo-induced apoptosis [16], indicating that Evo-induced apoptosis may have been due to an imbalance in ER resulting from opening of a calcium channel, but not TRPV1 activation.

Autophagy was found in neurons and glial cells in an ischemia-mimicking animal model [24], suggesting that glial cells may serve as one of the damage targets in stroke. 3-MA was shown to possess the ability to reduce the stroke-induced infarct volume, brain edema, and motor deficits [25, 26]. Furthermore, postischemic intracerebroventricular injections of 3-MA reduced the lesion volume after initiation of ischemia [27]. However, rapamycin and a GSK-3*β* inhibitor both activated autophagy to reduce ischemia-induced neuroinflammation and necrosis of neurons [13, 28]. An autophagy inhibitor reduced melatonin-mediated neuroprotection [15], suggesting that autophagy may play a survival role in response to ischemia-induced environmental stress. Therefore, the role of autophagy in stroke is controversial. The reasons for this difference may have been due to the chemical agents used, such as 3-MA and rapamycin, both of which are nonspecific inhibitors of autophagy. Thus, the effects resulting from blockade of autophagy using these agents must be carefully considered. In this study, we found that a natural compound, Evo, could activate JNK-mediated protective autophagy in astrocytes, suggesting that activation of TRPV1 may be a possible target for application in treating acute cerebral ischemia, but the detailed molecular mechanism and its significance in the clinic remain to be further investigated.

Disturbance of intracellular calcium levels was shown to be involved in regulating cell death. Our previous reports demonstrated that Evo could induce the release of calcium from the ER, which led to autophagy and apoptosis, since blockade of the calcium channel of ER resulted in reductions in apoptosis and autophagy [16]. In this study, we found that a scavenger of extracellular calcium and an inhibitor of TRPV1 both reduced Evo-induced intracellular calcium elevation and autophagy but increased the levels of apoptosis, suggesting that Evo-induced elevation of intracellular calcium may be due to release of calcium by ER and an influx of extracellular calcium. It was reported that a TRPV1 agonist could induce cell death through an ER stress pathway [8]. Furthermore, reduction of ER stress by an IP_3_R inhibitor decreased *β*-amyloid-induced neurotoxic effects on astrocytes [29], suggesting that Evo-induced apoptosis may result from induction of ER stress, but not an elevation of intracellular calcium. Collectively, the Evo-induced increase in intracellular calcium led to the induction of autophagy, which may be an adaptive response to protect astrocytes from cell death resulting from ER stress.

Currently, there is no effective therapy for cerebral ischemia, which causes cognitive and motor dysfunction, neurodegenerative diseases, and even acute death. In this study, we found that Evo, extracted from *E. rutaecarpa*, has the ability to induce survival autophagy in astrocytes through TRPV1-dependent signaling, which may provide a possible option for ischemia treatment.
